# Bridge, Reverse Bridge, and Their Control

**DOI:** 10.3390/e27070718

**Published:** 2025-07-02

**Authors:** Andrea Baldassarri, Andrea Puglisi

**Affiliations:** 1 Institute for Complex Systems CNR, University of Rome “La Sapienza”, P.le Aldo Moro 2, 00185 Rome, Italy; 2 Department of Physics, University of Rome “La Sapienza”, P.le Aldo Moro 2, 00185 Rome, Italy; 3 Istituto Nazionale Fisica Nucelare (INFN), Sezione Roma2, Via della Ricerca Scientifica 1, 00133 Rome, Italy

**Keywords:** time-reversal, stochastic bridge, reverse diffusion, brownian gyrators

## Abstract

We investigate the bridge problem for stochastic processes, that is, we analyze the statistical properties of trajectories constrained to begin and terminate at a fixed position within a time interval τ. Our primary focus is the time-reversal symmetry of these trajectories: under which conditions do the statistical properties remain invariant under the transformation t→τ−t? To address this question, we compare the stochastic differential equation describing the bridge, derived equivalently via Doob’s transform or stochastic optimal control, with the corresponding equation for the time-reversed bridge. We aim to provide a concise overview of these well-established derivation techniques and subsequently obtain a local condition for the time-reversal asymmetry that is specifically valid for the bridge. We are specifically interested in cases in which detailed balance is not satisfied and aim to eventually quantify the bridge asymmetry and understand how to use it to derive useful information about the underlying out-of-equilibrium dynamics. To this end, we derived a necessary condition for time-reversal symmetry, expressed in terms of the current velocity of the original stochastic process and a quantity linked to detailed balance. As expected, this formulation demonstrates that the bridge is symmetric when detailed balance holds, a sufficient condition that was already known. However, it also suggests that a bridge can exhibit symmetry even when the underlying process violates detailed balance. While we did not identify a specific instance of complete symmetry under broken detailed balance, we present an example of partial symmetry. In this case, some, but not all, components of the bridge display time-reversal symmetry. This example is drawn from a minimal non-equilibrium model, namely Brownian Gyrators, that are linear stochastic processes. We examined non-equilibrium systems driven by a "mechanical” force, specifically those in which the linear drift cannot be expressed as the gradient of a potential. While Gaussian processes like Brownian Gyrators offer valuable insights, it is known that they can be overly simplistic, even in their time-reversal properties. Therefore, we transformed the model into polar coordinates, obtaining a non-Gaussian process representing the squared modulus of the original process. Despite this increased complexity and the violation of detailed balance in the full process, we demonstrate through exact calculations that the bridge of the squared modulus in the isotropic case, constrained to start and end at the origin, exhibits perfect time-reversal symmetry.

## 1. Introduction

Stochastic processes are essential for describing natural phenomena that are significantly influenced by unpredictable forces [1]. Occurring across all space–time scales and within diverse scientific disciplines, they model Brownian motion of micrometric particles in fluids, reproduce the irregular or avalanche-like dynamics of electroencephalographic activity, and provide a useful description of stock-market prices, among other examples [2]. Remarkably, the same stochastic model can exhibit predictive power in seemingly disparate contexts [3]. This arises from the fact that noise often results from coarse-graining, which smooths out intricate physical details and isolates the essential aspects of a phenomenon [4]. Observations of noisy data can yield substantial information and serve as a valuable inference tool. For instance, the distance of a system from thermodynamic equilibrium [5] can be deduced from its fluctuations, as time-reversibility is expected to be encoded in their statistics [6,7]. However, while time-symmetry breaking can be evident in fluctuations, it may also become obscured due to the averaging out of crucial degrees of freedom [8].

This paper focuses on a specific tool within the theory of stochastic processes that is particularly useful in data analysis for discerning the presence or absence of time-reversal symmetry: the stochastic bridge. The term "bridge", in this context, refers to the ensemble of trajectories that start and end at the same specified point within a fixed time interval. This—apparently theoretical—quantity is similar to the so-called “average pulse”, “average avalanche”, or “average fluctuation” shape, which is measured in several experiments, e.g., in the physics of magnetic materials [9,10,11,12,13,14] or in general in materials science [15,16,17,18,19,20], geophysics [21,22,23,24], biology [25,26], neuroscience [27,28,29,30,31,32,32], and astrophysics [33,34]. In several cases, asymmetric shapes have been observed without a physical interpretation because of the lack of a general theory on the subject (the only exception is the asymmetry in the pulse shape observed in some ferromagnetic materials, which has been related to an effective negative mass effect [35,36]).

Intuitively, a statistically significant difference between a bridge and its time-reversed counterpart should indicate a violation of the underlying process’s time-reversal symmetry [37]. To rigorously examine the conditions under which this connection holds, we provide a self-contained development of bridge theory. This approach ensures that readers without prior knowledge of the subject can follow the derivation through to our central result, the symmetry condition presented in Equation (31). Our derivation relies on the formulation of the effective stochastic differential equation for the bridge, which can also be derived via optimal control (see for instance [38]). From that, we address the problem of bridge reverse diffusion. Reverse diffusion is a topic that has previously been introduced in physics in the context of stochastic mechanics (an alternative formulation of non-relativistic quantum mechanics [39]; see [40] for an introduction and [41] for a survey of these kinds of approaches) that is currently of significant interest in machine learning due to its fundamental role in techniques for reconstructing data from noise, as well as in generative models [42,43,44]. A careful comparison between the forward and reversed stochastic differential equations allows us to discuss the time-reverse symmetry of the process. We highlight that a natural sufficient condition for the symmetry of the bridge under time-reversal is detailed balance, as has already been discussed in the literature; see [37] for a enlightening historical summary.

As an instance of the theory, we consider a minimal stochastic model that closely reproduces the observed phenomenology of the asymmetric pulse shape [45] and at the same time is suitable for an analytic study [46]. The model, known as a Brownian Gyrator (BG) in the context of stochastic thermodynamics [47,48,49,50], can be regarded as an extension of the ABBM/CIR process, a paradigmatic model that was introduced to describe the phenomena that manifest “crackling noise” and that can be also considered as a kind of mean field theory for the problem of the dynamics of an elastic manifold in the presence of quenched pinning disorder [51].

We give, in Section 2, a recapitulation of the theory of continuous Markov processes (Fokker–Planck and stochastic differential equations). In Section 3, we describe bridge theory, and in Section 4, we describe how the same results have been obtained through optimal control theory. In Section 5, we recall the problem of reverse diffusion, and at the end of that section, we apply the corresponding theory to bridge statistics in order to deduce the necessary condition for bridge time-symmetry (this is our original contribution). Finally, in Section 6, we list a series of examples and applications. Conclusions are drawn in Section 7.

## 2. Continuous Markov Processes

We consider a stochastic process x(t) that is a Markov process, i.e., a process fully characterized by the distribution P(x,t) and the propagator $P(x,t|x_{0},t_{0})$, which represent the probability of observing the value *x* at time *t* without or with, respectively, the knowledge of a value $x_{0}$ at a past time $t_{0}$. Every other probability can be evaluated by means of P(x,t) and $P(x,t|x_{0},t_{0})$ only, since the probabilities of arbitrary *n* values at times $t_{1}<t_{2}<...<t_{n-1}<t_{n}$ are defined as follows:

$$P\left(x_{n},t_{n};x_{n-1},t_{n-1};...;x_{1},t_{1}\right)=P\left(x_{n},t_{n}|x_{n-1},t_{n-1}\right)...P\left(x_{2},t_{2}|x_{1},t_{1}\right)P\left(x_{1},t_{1}\right).$$

The Markov property implies that the propagator fulfills the following closed integral relation, known as the Chapman–Kolmogorov (CK) equation:

(1) $$P\left(x,t|x_{0},t_{0}\right)=\int dyP\left(x,t|y,\tau \right)P\left(y,\tau |x_{0},t_{0}\right),$$

where τ is an intermediate time $t_{0}<\tau <t$, while at the extremes $\tau =t_{0}$ and τ=t, the relation is trivial, considering that, from the very definition of the propagator, P(x,t|y,t)=δ(x−y).

An important class of Markov processes are the continuous processes. The prototype of this class is the Wiener process w(t), which has a Gaussian propagator

$$P_{0}\left(w,t|w_{0},t_{0}\right)=P_{0}\left(w-w_{0},t-t_{0}|0,0\right)=g_{t-t_{0}}\left(w-w_{0}\right),$$

where

$$g_{s}\left(x\right)={\left(2\pi s\right)}^{-1/2}\exp\left[-x^{2}/\left(2s\right)\right],$$

whose variance increase linearly from $t-t_{0}$, resulting in a linear time correlation for the process values, as follows:

$$\langle \left[w\left(t\right)-w\left(t_{0}\right)\right]\left[w\left(t\right)-w\left(t_{0}\right]\right)\rangle =\left|t-t_{0}\right|.$$

(Here, we restrict the discussion to the one-dimensional case, with x(t) and w(t) belonging to the real axis, but the *n*-dimensional case can be similarly defined). It is easy to show that the Gaussian propagator of the Wiener process satisfies the CK Equation (1), essentially because of the stability of the Gaussian distribution under convolution.

However, it is easy to verify that the Wiener propagator satisfies a much more simpler and more useful equation, as follows:

(2) $$\partial_{t}P_{0}\left(z,t|0,0\right)=\partial_{z}^{2}P_{0}\left(z,t|0,0\right).\;$$

The partial differential Equation (2) is much more useful than Equation (1), which is a non-linear integral equation, but it is valid for the Wiener process only. One thus may wonder how we can generalize Equation (2) to all continuous Markov processes.

A heuristic general definition for this class of processes reads as follows:

(3) x(t+dt)=x(t)+a(x(t),t)dt+b(x(t),t) dw(t),

where a(x,t) is a deterministic drift flow, b(x(t),t) is an amplitude factor that rules the relative impact of the random noise on the process, and dw(t) is the infinitesimal increment of the Wiener process representing the noise. A heuristic derivation of the corresponding forward Fokker–Planck equation follows [40].

Consider P(y,t+dt|x,t): the probability of obtaining *y* at time t+dt, knowing *x* at time *t* is equivalent to the probability of obtaining the right Wiener kick dw(t)=(y−x−a(x,t))/b(x,t) according to Equation (3):

(4) $$P\left(y,t+dt|x,t\right)=P_{0}\left[\left(y-x-a\left(x,t\right)dt\right)/b\left(x,t\right),dt|0,0\right]$$

Subtracting $\delta \left(y-x\right)=P\left(y,t|x,t\right)=P_{0}\left(y,t|0,0\right)$ and dividing for dt both sides, we obtain the following in the limit for dt→0 on the left hand side of Equation (4):

$$\partial_{t^{\prime }}P\left(y,t^{\prime }|x,t\right){|}_{t^{\prime }=t}.$$

For the right side, we obtain, developing for small dt:

$$\frac{1}{dt}\left\{P_{0}\left[\left(y-x\right)/b\left(x,t\right),dt|0,0\right]+\partial_{y}P_{0}\left[\left(y-x\right)/b\left(x,t\right),dt|0,0\right]\frac{a(x,t)}{b(x,t)}dt-\delta \left(x-y\right)\right\}.$$

This gives, in the limit dt→0:

$$\partial_{t^{\prime }}P_{0}\left[\left(y-x\right)/b\left(x,t\right),t^{\prime }|0,0\right]{|}_{t^{\prime }=0}+\frac{a(x,t)}{b(x,t)}\partial_{y}\delta \left[\left(y-x\right)/b\left(x,t\right)\right].$$

Now, using

$$\frac{1}{c}\delta \left[z/c\right]=\delta \left[z\right],$$

and Equation (2), noting that

$$\partial_{z}^{2}P_{0}\left(z,t^{\prime }|0,0\right){|}_{z=(y-x)/b(x,t)}=b^{2}\left(x,t\right)\partial_{y}^{2}P_{0}\left[\left(y-x\right)/b\left(x,t\right),t^{\prime }|0,0\right],$$

we finally transform Equation (4) as follows:

(5) $$\partial_{t^{\prime }}P\left(y,t^{\prime }|x,t\right){|}_{t^{\prime }=t}=a\left(x,t\right)\partial_{y}\delta \left[y-x\right]+\frac{1}{2}b{\left(x,t\right)}^{2}\partial_{y}^{2}\delta \left[y-x\right]\;$$

In this way, we obtained an expression for the time derivative of the propagator of the Markov process at equal times that we can use in the differentiated CK equation in the limit τ→t, as follows:

(6) $$\partial_{t}P\left(x,t|x_{0},t_{0}\right)=\int dy\partial_{t}P\left(x,t|y,\tau \right)P\left(y,\tau |x_{0},t_{0}\right).$$

Using integration by parts (neglecting border terms, which vanish in general), we obtain the so called forward Fokker–Planck (FFP) equation for a (continuous) Markov stochastic process:

(7) $$\partial_{t}P\left(x,t|x_{0},t_{0}\right)=-\partial_{x}\left[a\left(x,t\right)P\left(x,t|x_{0},t_{0}\right)\right]+\frac{1}{2}\partial_{x}^{2}\left[b{\left(x,t\right)}^{2}P\left(x,t|x_{0},t_{0}\right)\right].$$

This interesting derivation hides an important hypothesis, though. In our derivation, we used the stochastic Equation (3), which relates the increment of the Markov process x(t) to the Wiener process w(t). In doing so, we decided that the value of the process at time t+dt does not depend on the values of the Wiener process at times larger than *t*. This is the natural way to proceed, as, for instance, when one wants to produce a stochastic sampling of the stochastic trajectories (the Euler–Maruyama method).

In fact, when one tries to provide a more correct foundation in the initial stochastic differential equation (sde), Equation (3), one discovers that it is well defined only if *b* does not depend on *x*. The correct procedure by which to give a mathematical meaning to the general sde is to define its integral version and then give a precise definition of the stochastic integral $\int_{0}^{t}b\left(x\left(t^{\prime }\right),t^{\prime }\right)dw\left(t^{\prime }\right)$, which is possible via a partition of the time-integration interval $0,t_{1},t_{2},...,t_{N}$ and an arbitrary choice of the time at which the function b(x(t),t) is computed inside the infinitesimal interval $\left[t_{i},t_{i+1}\right]$: if one chooses the beginning of the interval $t=t_{i}$ (Ito scheme), the previous derivation gives the correct result. A general choice where *b* is computed at point $\left(1-\gamma \right)x\left(t_{i}\right)+\gamma x\left(t_{i+1}\right)$ gives the following γ-FFP equation [52]:

$$\partial_{t}P=-\partial_{x}\left[(a\left(x,t\right)+\gamma b\left(x,t\right)\partial_{x}b\left(x,t\right))P\right]+\frac{1}{2}\partial_{x}^{2}\left[b^{2}\left(x,t\right)P\right],$$

where we use the abbreviated form $P=P(x,t|x_{0},t_{0})$. Two interesting cases are the Stratonovich case γ=1/2, in which the FFP can be rewritten as

$$\partial_{t}P=-\partial_{x}\left[a(x,t)P\right]+\frac{1}{2}\partial_{x}\left[b\left(x,t\right)\partial_{x}b\left(x,t\right)P\right],$$

and the anti-Ito or isothermal case γ=1, in which the FFP can be rewritten as

$$\partial_{t}P=-\partial_{x}\left[a(x,t)P\right]+\frac{1}{2}\partial_{x}\left[b^{2}\left(x,t\right)\partial_{x}P\right].$$

Below, unless otherwise specified, we will adopt the Ito integration scheme, that is, the γ-FFP equation for γ=0.

The FFP equation is a continuity equation for the probability, since

$$\partial_{t}P=-\partial_{x}\left(VP\right),\;$$

where

(8) $$V=a-\frac{1}{2P}\partial_{x}\left(b^{2}P\right)$$

is the current velocity and J=VP is the probability current. The deterministic case b=0 corresponds to the Liouville equation. When b≠0, the new term, sometimes called osmotic velocity $U=\frac{1}{2P}\partial_{x}\left(b^{2}P\right)$, manifests the effect of random fluctuations.

A stationary solution of the FFP may arise if it exists a stationary distribution $P_{s}$ such that $\partial_{x}\left(V_{s}P_{s}\right)=0$. A special case arises when the current velocity is zero V=0, that is, when

(9) $$2a-\partial_{x}b^{2}-b^{2}\partial_{x}\log P_{s}=0,$$

which is equivalent (see [2]) to the condition of detailed balance for the stationary process. More precisely, for the stationary process, defined by the stationary one and two point distributions $P\left(x,t\right)=P_{s}\left(x\right)$, and $P\left(x_{1},t_{1};x_{2},t_{2}\right)=P_{s}\left(x_{1},t_{1};x_{2},t_{2}\right)\equiv P\left(x_{1},t_{1}|x_{2},t_{2}\right)P_{s}\left(x_{2}\right)$, the detailed balance condition reads:

(10) $$P_{s}\left(x_{1},t_{1};x_{2},t_{2}\right)=P_{s}\left(x_{2},t_{1};x_{1},t_{2}\right),$$

stating the invariance of the process under time reversal. (Here, we are considering the simplest case of variables with even parity under time reversal. To better frame the general case, see [53]).

We recall that the propagator $P(x,t|x_{0},t_{0})$ can also be seen as a function of the conditioning variables $\hat{P}\left(x_{f},t_{f}|x,t\right)$, abbreviated as $\hat{P}$, which satisfies the so-called backward Fokker–Planck (BFP) equation, as follows:

(11) $$\partial_{t}\hat{P}=-a\left(x,t\right)\partial_{x}\hat{P}-\frac{1}{2}b^{2}\left(x,t\right)\partial_{x}^{2}\hat{P}.$$

*En passant*, we observe that the FFP and the BFP can be written as $\partial_{t}P=L_{F}P$ and $\partial_{t}\hat{P}=-L_{B}\hat{P}$, respectively. By simple integration by parts, it is easily seen that the two operators are adjoints, i.e., $L_{B}=L_{F}^{+}$, where the adjoint operation is defined through a scalar product $\left(f,Lg\right)=\left(L^{+}f,g\right)$ and (f,g)=∫dxf(x)g(x).

In the general dimension *d*, one has the Ito sde with independent Wiener processes whose increments are denoted by $dW_{i}$, i∈[1,d]

$$dx_{i}=a_{i}\left(x,t\right)dt+b_{i,j}\left(x,t\right)dW^{j}.$$

giving place to the d-dimensional FFP and the corresponding BFP

(12) $$\begin{array}{ccc} \partial_{t}P & = & -\partial_{x_{i}}\left[A_{i}P\right]+\frac{1}{2}\partial_{x_{i}}\partial_{x_{j}}\left[B_{i,j}P\right] \end{array}$$

(13) $$\begin{array}{ccc} \partial_{t}\hat{P} & = & -A_{i}\partial_{x_{i}}\hat{P}-\frac{1}{2}B_{i,j}\partial_{x_{i}}\partial_{x_{j}}\hat{P} \end{array}$$

where, here and below, we use $A_{i}=a_{i}$ and $B_{i,j}=b_{i,k}b^{k,j}$ (summation on repeated index is implied, while the position of the index and sub- or superscript is arbitrarily chosen just for clarity of typesetting).

Correspondingly, the current and the osmotic velocities read, respectively, as follows:

(14) $$\begin{array}{ccc} V_{i} & = & A_{i}-\frac{1}{2P}\partial_{x_{j}}\left[B_{i,j}P\right] \end{array}$$

(15) $$\begin{array}{ccc} U_{i} & = & \frac{1}{2P}\partial_{x_{j}}\left[B_{i,j}P\right], \end{array}$$

while the detailed balance keeps the form given in Equation (10).

## 3. Bridge

The bridge of a stochastic process is defined as the set of trajectories passing through a value $x_{0}$ at time $t_{0}$ and a value $x_{f}$ at later time $t_{f}$. A more general definition was introduced in physics by Schrödinger [37,54], who proposed the problem of a stochastic process with the starting and final distribution fixed. This problem, known as Schrödinger bridge, was later connected and well framed in terms of optimal transport and control theory (see [55] for an introduction and [56] for a survey), giving a better understanding of the clever solution found by Schröedinger. In this paper, we will not address this general problem, and we chose to use only the simplest definition of stochastic bridge, with fixed initial and final values (which correspond to the very special case of degenerate delta-like initial and final distributions).

In the case of a continuous Markov process, the subset of trajectories defining the bridge represents a novel continuous Markov process. Its distribution is defined as follows:

$$P_{B}\left(x,t\right)=P\left(x,t|x_{0},t_{0};x_{f},t_{f}\right)=\frac{P\left(x,t|x_{0},t_{0}\right)P\left(x_{f},t_{f}|x,t\right)}{P(x_{f},t_{f}|x_{0},t_{0})}.$$

Note that the double conditioning in $P(x,t|x_{0},t_{0};x_{f},t_{f})$ is not in contradiction with the Markovianity of the original process, since $t_{0}<t<t_{f}$. It is possible to derive the differential equation that governs the evolution of $P_{B}\left(x,t\right)$, making use of both the FFP and the BFP of the original problem [57].

We will show below that $P_{B}\left(x,t\right)$ satisfies a modified FFP where the diffusion coefficients are the same $B^{i,j}$ that were used in the original process, while the drift coefficients are modified by the following terms:

$$A^{i}\to A^{i}+B^{i,j}\partial_{x_{j}}\log P\left(x_{f},t_{f}|x,t\right).$$

The proof proceeds with time-deriving $P_{B}$ and then uses Equations (12) and (13) as follows:

(16) $$\partial_{t}P_{B}=\frac{1}{P†}\left\{-\partial_{x_{i}}\left[A^{i}P\right]\hat{P}+\frac{1}{2}\partial_{x_{i}}\partial_{x_{j}}\left[B^{i,j}P\right]\hat{P}-PA^{i}\partial_{x_{i}}\hat{P}-\frac{1}{2}PB^{i,j}\partial_{x_{i}}\partial_{x_{j}}\hat{P}\right\},$$

where $P†=P(x_{f},t_{f}|x_{0},t_{0})$ is the normalization, which is constant with respect to the *x* derivatives. We note that the first and the third terms in Equation (16) are enough to build a first drift term, as follows:

$$-\partial_{x_{i}}\left[A^{i}P_{B}\right].$$

Then, we note that

$$\frac{1}{2}\partial_{x_{i}}\partial_{x_{j}}\left[B_{i,j}P\hat{P}\right]=\frac{1}{2}\partial_{x_{i}}\partial_{x_{j}}\left[B^{i,j}P\right]\hat{P}+\frac{1}{2}B^{i,j}P\partial_{x_{i}}\partial_{x_{j}}\hat{P}+\partial_{x_{i}}\left[B^{i,j}P\right]\partial_{x_{j}}\hat{P},$$

which allows us to rewrite the second and fourth term in Equation (16) as

(17) $$\frac{1}{2}\partial_{x_{i}}\partial_{x_{j}}\left[B_{i,j}P\hat{P}\right]-PB^{i,j}\partial_{x_{i}}\partial_{x_{j}}\hat{P}-\partial_{x_{i}}\left[B^{i,j}P\right]\partial_{x_{j}}\hat{P}.$$

Now we can observe that since

$$\partial_{x_{i}}\left[P\hat{P}B^{ij}\partial_{x_{j}}\log\hat{P}\right]=\partial_{x_{i}}\left[PB^{i,j}\partial_{x_{j}}\hat{P}\right]=\partial_{x_{i}}\left[B^{i,j}P\right]\partial_{x_{j}}\hat{P}+B^{i,j}P\partial_{x_{i}}\partial_{x_{j}}\hat{P},$$

we have demonstrated that the terms mentioned before contribute to the sum of a diffusion term for $P_{B}$ and a new drift term. All of these together give the new FFP for $P_{B}$:

$$\partial_{t}P_{B}\left(x,t\right)=\frac{1}{2}\partial_{x_{i}}\partial_{x_{j}}\left[B^{i,j}\left(x,t\right)P_{B}\left(x,t\right)\right]-\partial_{x_{i}}\left[\left(A\left(x,t\right)+B^{i,j}\left(x,t\right)\partial_{x_{j}}\log P\left(x_{f},t_{f}|x,t\right)\right)P_{B}\left(x,t\right)\right]$$

with initial condition $P_{B}\left(x,0\right)=\delta \left(x-x_{0}\right)$.

Since the bridge is a Markov process, its propagator $P_{B}\left(x,t|y,t^{\prime }\right)$ connecting $y,t^{\prime }$ to x,t (with $t_{0}\leq t^{\prime }\leq t<t_{f}$) satisfies the same equation of $P_{B}\left(x,t\right)$ with an “initial” condition $P_{B}\left(x,t^{\prime }|y,t^{\prime }\right)=\delta \left(x-y\right)$.

The trajectories of the continuous Markov process defined by $P_{B}\left(x,t\right)$ and $P_{B}\left(x,t|y,t^{\prime }\right)$ are described by the Ito sde as follows:

(18) $$dx_{B}^{i}=\left[A^{i}\left(x_{B},t\right)+B^{i,j}\left(x_{B},t\right)\partial_{x_{B}^{j}}\log P\left(x_{f},t_{f}|x_{B},t\right)\right]dt+b^{i,j}\left(x_{B},t\right)dW^{j}\left(t\right),$$

Note that the term $P(x_{f},t_{f}|x,t)$ in the sde is the propagator of the unconditioned process whose bridge statistics we want to compute. Usually, we are interested in the bridge statistics for the stationary process, so here and below we assume that $P\left(x,t|y,t^{\prime }\right)=P\left(x,t-t^{\prime }|y,0\right)=P_{s}\left(x,t-t^{\prime };y,0\right)/P_{s}\left(y\right)$ and $P_{s}\left(x\right)=\lim_{t\to \infty }P\left(x,t|y,0\right)$.

## 4. Bridge via Stochastic Control

Here, we recall how to recover the previous result in the framework of stochastic control [58,59]. We provide a generalization of the process described in [38]; see also [60]. Let us start with a controlled sde:

(19) $$dx_{i}=a_{i}\left(x,t\right)dt+b_{i,j}\left(x,t\right)dW_{j}+u_{i}\left(x,t\right)dt,$$

where the control term u(x,t) has to be determined by minimizing the cost function

(20) $$J\left(x,t_{f}\right)=\int_{0}^{t_{f}}\left[\frac{1}{2}u_{i}\left(x,t\right){\left[\beta^{-1}\left(x,t\right)\right]}_{i,j}u_{j}\left(x,t\right)\left(x,t\right)+\sum_{i}\Omega_{i}\left(x,t\right)\right]dt+z_{t_{f}}\left(x\right).$$

The first integral on the right-hand side is the sum of the running cost of the process (specified by Ω) and that of the control. The quadratic form of the running cost for the control will be justifies below, where we show that this choice select the specific sample of free trajectories in order to give the correct final result. We also introduce the arbitrary function β(x,t), which is a positive definite matrix; we will show what choice is necessary to obtain the final result. The term $z_{t_{f}}\left(x\right)$ is a cost on the final position of the trajectories that will be used to impose the arrival point of the bridge.

In order to solve the control problem, one can proceed with the Hamilton–Jacobi–Bellman (HJB) equation, which is derived from the so called dynamical programming principle:

(21) $$\partial_{t}V+\min_{u}\left\{\frac{1}{2}u_{i}{\left[\beta^{-1}\right]}_{i,j}u_{j}+\left(a_{i}+u_{i}\right)\partial_{x_{i}}V+\frac{1}{2}B_{i,j}\partial_{x_{i}}\partial_{x_{j}}V+\Omega_{i}\right\}=0$$

(we omitted the dependence on x,t in the notation), where $B_{i,j}=b_{i,k}b_{k,j}$. The minimum is achieved for the value of $u_{i}$ given by: ${\left[\beta^{-1}\right]}_{i,j}u_{j}+\partial_{x_{i}}V=0$, that is

$$u_{i}=-\beta_{i,j}\partial_{x_{j}}V,$$

which, as introduced in the HJB Equation (21), gives the non-linear partial differential equation

$$\partial_{t}V+\left\{-\frac{1}{2}\beta_{i,j}\partial_{x_{i}}V\partial_{x_{j}}V+a_{i}\partial_{x_{i}}V+\frac{1}{2}B_{i,j}\partial_{x_{i}}\partial_{x_{j}}V+\Omega_{i}\right\}=0,$$

to be solved with the final condition $V\left(x,t_{f}\right)=z_{t_{f}}\left(x\right)$.

We get rid of the non-linear terms $\partial_{x_{i}}V\partial_{x_{j}}V$ by means of the transformation W=ψ(V):

$$\partial_{t}W=-a_{i}\partial_{x_{i}}W-\frac{1}{2}B_{i,j}\partial_{x_{i}}\partial_{x_{j}}W+\psi^{\prime }\left(V\right)\Omega_{i}-\frac{1}{2}\left(\beta_{i,j}\psi^{\prime }\left(V\right)+B_{i,j}\psi^{\prime \prime }\left(V\right)\right)\partial_{x_{i}}V\partial_{x_{j}}V,$$

and the choice of the Cole–Hopf transformation ψ(V)=exp(−V) with β=B.

Moreover, from now on, we focus on the case Ω=0, since we are interested in the bridge of the process without any running conditioning. In this way, we obtain the following equation for *W*:

$$\partial_{t}W=-a_{i}\partial_{x_{i}}W-\frac{1}{2}B_{i,j}\partial_{x_{i}}\partial_{x_{j}}W,$$

which is the BFP of the original process.

However, we have to impose the final boundary conditions given by the final cost $W\left(x,t_{f}\right)=\exp\left(-z_{t_{f}}\left(x\right)\right)$. Using the Green function of the equation, which corresponds to the free propagator of the process (since P(y,t|x,t)=δ(x−y)), we obtain the following:

$$W\left(x,t\right)=\int P\left(y,t_{f}|x,t\right)\exp\left(-z_{t_{f}}\left(y\right)\right)dy$$

In order to impose the final position of the bridge $x\left(t_{f}\right)=x_{f}$, we can choose an exponential cost $z_{t_{f}}\left(x\right)=\exp\left[-F|x-x_{f}{|}^{2}\right]$ in the limit of F≫1, which in the Laplace approximation gives the following:

$$W\left(x,t\right)\approx \sqrt{\frac{\pi }{F}}\left\{P\left(x_{f},t_{f}|x,t\right)+O\left(1/F\right)\right\},$$

This finally results in the control, as follows:

$$u_{i}\left(x,t\right)=B_{i,j}\partial_{x_{j}}\log P\left(x_{f},t_{f}|x,t\right),$$

which leads to Equation (18).

## 5. Reverse Diffusion

The problem of reverse diffusion can be simply stated as follows [61]: imagine a continuous Markov stochastic process defined by an initial distribution $P\left(x,t_{0}\right)=P_{0}\left(x\right)$ and its propagator $P(x,t|x_{1},t_{1})$, with $t_{0}<t_{1}<t$, where both the propagator and $P\left(x,t\right)=\int dyP\left(x,t|y,t_{0}\right)P_{0}\left(y\right)$ solve the FFP (12). Now, we consider the probability of a value x(t) conditioned to the future value $x\left(t_{f}\right)=x_{f}$ (i.e., $t<t_{f}$): $P(x,t|x_{f},t_{f})$. We want to answer the question, “Is there an sde (and therefore a corresponding FFP) satisfied by this process?”.

The correct way to answer this question follows. First, one can look for an equation for the joint probability $P(x,t;x_{f},t_{f})$. This can be done considering the following:

$$\partial_{t}P\left(x,t;x_{f},t_{f}\right)=\partial_{t}\left[P\left(x_{f},t_{f}|x,t\right)P\left(x,t\right)\right]=P\left(x,t\right)\partial_{t}P\left(x_{f},t_{f}|x,t\right)+P\left(x_{f},t_{f}|x,t\right)\partial_{t}P\left(x,t\right).$$

Then, one has to use the BFP equation to express $\partial_{t}P\left(x_{f},t_{f}|x,t\right)$ and the FFP to express $\partial_{t}P\left(x,t\right)$. In this way, we obtain an expression $\partial_{t}P\left(x,t;x_{f},t_{f}\right)$ as a function of partial derivatives in *x* of P(x,t) and $P(x_{f},t_{f}|x,t)$. If now, one reintroduces the joint probability, writing $P\left(x_{f},t_{f}|x,t\right)=P\left(x_{f},t_{f};x,t\right)/P\left(x,t\right)$, one obtains, after algebraic manipulation, an equation for the joint probability, as follows:

(22) $$\begin{array}{c} \partial_{t}P\left(x,t;x_{f},t_{f}\right)=-\partial_{x_{i}}\left\{\left(A^{i}\left(x,t\right)-\frac{1}{P(x,t)}\partial_{x_{j}}\left[B^{i,j}\left(x,t\right)P\left(x,t\right)\right]\right)P\left(x,t;x_{f},t_{f}\right)\right\}-\\ \frac{1}{2}\partial_{x_{i}}\partial_{x_{j}}\left[B^{i,j}\left(x,t\right)P\left(x,t;x_{f},t_{f}\right)\right]. \end{array}$$

Dividing both terms for $P(x_{f},t_{f})$ one obtains a new equation for $P(x,t|x_{f},t_{f})$:

(23) $$\begin{array}{c} \partial_{t}P\left(x,t|x_{f},t_{f}\right)=-\partial_{x_{i}}\left\{\left(A^{i}\left(x,t\right)-\frac{1}{P(x,t)}\partial_{x_{j}}\left[B^{i,j}\left(x,t\right)P\left(x,t\right)\right]\right)P\left(x,t|x_{f},t_{f}\right)\right\}\\ -\frac{1}{2}\partial_{x_{i}}\partial_{x_{j}}\left[B^{i,j}\left(x,t\right)P\left(x,t|x_{f},t_{f}\right)\right]. \end{array}$$

Note that Equation (23) is neither an FFP (because of the signs before the diffusion term) nor a BFP (except in the case of *A* and *B* bring independent of *x*), and we can not directly derive a corresponding sde from that.

A simple interpretation arises from considering a change in the time variable $t^{\prime }=t_{f}-t$. Setting $t_{0}=0$, without loss of generality, the new variable varies between 0 and $t_{f}$, and obviously, $\partial_{t}=-\partial_{t^{\prime }}$. Naming $P^{R}\left(x,t^{\prime }|x_{f},0\right)=P\left(x,t_{f}-t^{\prime }|x_{f},t_{f}\right)$ allows one to rewrite Equation (23) as follows:

(24) $$\begin{array}{c} \partial_{t^{\prime }}P^{R}\left(x,t^{\prime }|x_{f},0\right)=\\ -\partial_{x_{i}}\left\{\left(\frac{1}{P(x,t_{f}-t^{\prime })}\partial_{x_{j}}\left[B^{i,j}\left(x,t_{f}-t^{\prime }\right)P\left(x,t_{f}-t^{\prime }\right)\right]-A^{i}(x,t_{f}-t^{\prime }\right)P^{R}\left(x,t^{\prime }|x_{f},0\right)\right\}\\ +\frac{1}{2}\partial_{x_{i}}\partial_{x_{j}}\left[B^{i,j}\left(x,t_{f}-t^{\prime }\right)P^{R}\left(x,t^{\prime }|x_{f},0\right)\right] \end{array}$$

Again, this equation can be used to define a (non-homogeneous) continuous Markov process with a single time distribution $P^{R}\left(x,t|x_{f},0\right)$ and a propagator satisfying the same equation:

(25) $$\begin{array}{c} \partial_{t}P^{R}\left(x,t|y,t^{\prime }\right)=-\partial_{x_{i}}\left\{{\bar{A}}_{i}\left(x,t_{f}-t\right)P^{R}\left(x,t|y,t^{\prime }\right)\right\}+\frac{1}{2}\partial_{x_{i}}\partial_{x_{j}}\left[B^{i,j}\left(x,t_{f}-t\right)P^{R}\left(x,t|y,t^{\prime }\right)\right] \end{array}$$

with

(26) $${\bar{A}}_{i}\left(x,t\right)=\left(\frac{1}{P(x,t)}\partial_{x_{j}}\left[B^{i,j}\left(x,t\right)P\left(x,t\right)\right]-A^{i}\left(x,t\right)\right)$$

and initial condition $P^{R}\left(x,t^{\prime }|y,t^{\prime }\right)=\delta \left(x-y\right)$. The trajectories of this continuous Markov process are described by the sde as follows:

(27) $$d{\bar{x}}^{i}={\bar{A}}_{i}\left(\bar{x},t_{f}-t\right)dt+b^{i,j}\left(\bar{x},t_{f}-t\right)dW_{t}^{j},$$

where now W(t) is a usual (Ito) Wiener process. The starting condition $\bar{x}\left(0\right)$ of this sde must be chosen with probability $P_{R}\left(\bar{x},0\right)=P\left(\bar{x},t_{f}\right)$. Obviously, if one considers the reverse of the reversed process, one recovers the forward process.

Note that the new drift can be expressed as

(28) $${\bar{A}}_{i}\left(x,t\right)=U_{i}\left(x,t\right)-V_{i}\left(x,t\right)$$

which is the the osmotic velocity of the original process $U_{i}=\frac{1}{2P}\partial_{x_{j}}\left[B_{i,j}P\right]$ minus its current velocity $V_{i}=A_{i}-\frac{1}{2P}\partial_{x_{j}}\left[B_{i,j}P\right]$. Note that, on the other hand, the drift of the original (forward) process could also be written in terms of V and U, as $A_{i}\left(x,t\right)=U_{i}\left(x,t\right)+V_{i}\left(x,t\right)$.

We also observe that, at odds with the forward process, in the reverse process we have to fix the starting condition as $Prob\left(\bar{x}\left(0\right)\right)=P\left(x,t_{f}\right)$ and the following evolution is driven by “controlled dynamics”, where a control term (the osmotic velocity) imposing at all times $Prob\left[x<\bar{x}\left(t\right)<x+dx\right]=P\left(x,t_{f}-t\right)dx$ is added to a reversed drift (opposite sign and $t\to t_{f}-t$) [62].

### 5.1. Stationary Reverse Process

The process defined by Equation (27) is defined with initial condition $Prob\left(x<\bar{x}\left(0\right)<x+dx\right)=P\left(x,t_{f}\right)dx$, and the ensemble of the trajectories satisfies $Prob\left(x<\bar{x}\left(t\right)=x\right)dx=P\left(x,t_{f}-t\right)dx$ until time $t=t_{f}$. P(x,t) is a one-time distribution probability of the (forward) process, i.e., it is governed by a sde with drift A(x,t) and diffusion term b(x,t). If we consider the case of a time-homogeneous process, that is, one where *A* and *b* do not depend on time, that admits a stationary distribution, we can consider the limit $t_{f}\to \infty$. In other words, we wonder what the reversed process of a stationary process is. From Equation (27), its sde is given as follows:

$$d{\bar{x}}^{i}=\left\{\frac{1}{P_{s}\left(\bar{x}\right)}\partial_{{\bar{x}}_{j}}\left[B^{i,j}\left(\bar{x}\right)P_{s}\left(\bar{x}\right)\right]-A^{i}\left(\bar{x}\right)\right\}dt+b^{i,j}\left(\bar{x}\right)dW_{t}^{j}.$$

The trajectory of a stationary process that is invariant under time reversal should have the same statistics as its reversed counterpart. This is achieved when the respective sdes coincide, that is, when

$$A^{i}\left(x\right)=\frac{1}{P_{s}\left(x\right)}\partial_{x_{j}}\left[B^{i,j}\left(x\right)P_{s}\left(x\right)\right]-A^{i}\left(x\right)$$

which means

$$V_{s}^{i}=0,$$

that is, when the stationary velocity current is zero. The vanishing of the stationary current implies zero entropy production, consistent with the requirement of time-reversal invariance [5].

### 5.2. Reverse Bridge

Now, we proceed to compute the reversed process of the bridge. In this case, we have to consider Equation (28) with the current and osmotic velocity of the bridge. Since we are considering the bridge of a stationary process defined by a $P\left(x,t\right)=P_{s}\left(x\right)$ and $P\left(x,t|y,t^{\prime }\right)=P_{s}\left(x,t-t^{\prime }|x,0\right)$, the distribution to be used in the expressions for the osmotic and current velocity is

$$P_{B}\left(x,t\right)=\frac{P_{s}\left(x,t|x_{0},0\right)P_{s}\left(x_{f},t_{f}-t|x,0\right)}{P_{s}\left(x_{f},t_{f}|x_{0},0\right)}.$$

while the drift of the bridge sde is

$$A_{B}^{i}\left(x,t\right)=A^{i}\left(x\right)+B^{i,j}\left(x\right)\partial_{x_{j}}\log P_{s}\left(x_{f},t_{f}-t|x,0\right)$$

Thus, the drift of the reverse bridge is as follows:

(29) $$\begin{array}{ccc} {\bar{A}}_{B}^{i}\left(x,t\right) & = & \frac{1}{P_{B}\left(x,t\right)}\partial_{x_{j}}\left[B^{i,j}\left(x\right)P_{B}\left(x,t\right)\right]-A_{B}^{i}\left(x,t\right) \end{array}$$

(30) $$\begin{array}{ccc} & = & -A^{i}\left(x\right)+\partial_{x_{j}}B^{i,j}\left(x\right)+B^{i,j}\left(x\right)\partial_{x_{j}}\log P_{s}\left(x,t|x_{0},0\right). \end{array}$$

Now, we consider the case $x_{0}=x_{f}$ and compute the difference between the forward and reversed drift, which is a measure of the bridge asymmetry with respect to the time reversal $t\to t_{f}-t$:

(31) $$\Delta^{i}\left(x,t\right)=A_{B}^{i}\left(x,t\right)-{\bar{A}}_{B}^{i}\left(x,t_{f}-t\right)=2A^{i}\left(x\right)-\partial_{x_{j}}B^{i,j}\left(x\right)+B^{i,j}\left(x\right)\partial_{x_{j}}\log\frac{P_{s}\left(x_{0},t_{f}-t|x,0\right)}{P_{s}\left(x,t_{f}-t|x_{0},0\right)}$$

that can be also expressed as follows:

(32) $$\Delta^{i}\left(x,t\right)=2V_{s}^{i}\left(x\right)+B^{i,j}\left(x\right)\partial_{x_{j}}\log\frac{P_{s}\left(x_{0},t_{f}-t;x,0\right)}{P_{s}\left(x,t_{f}-t;x_{0},0\right)}$$

where we have inserted the stationary joint distribution $P_{s}\left(x,t;y,0\right)=P\left(x,t|y,0\right)P_{s}\left(y\right)$ and $V_{s}\left(x\right)$ is the stationary current velocity of the original process:

$$V_{s}^{i}\left(x\right)=A^{i}\left(x\right)-\frac{1}{2}\partial_{x_{j}}B^{i,j}\left(x\right)-\frac{1}{2}B^{i,j}\left(x\right)\partial_{x_{j}}\log P_{s}\left(x\right).$$

Again, note that if the process satisfies detailed balance, both terms in Equation (32) vanish; see [37] for a review of the literature where this natural sufficient condition has been previously discussed. A necessary condition is, however, more important: it may happen that, even in the case of zero asymmetry, the two terms are (necessarily) both different from zero but their sum is equal to zero.

## 6. Examples for Asymmetry of Bridges

In this section, we compute the asymmetry in some simple processes. First, we consider a generic linear process; then, we consider the two-dimensional case, in particular the so-called Brownian Gyrator (BG), which has been introduced as a minimal model for a heat engine at the nano-scale [47,48,49,50]. In fact, we consider two versions that are not always equivalent: a thermal BG, where the engine is powered by two thermal baths at different temperatures, and a mechanical BG, where a single thermal bath is present, but a non-conservative force feeds the engine. The BG is a Gaussian process and therefore is analytically solvable but can sometimes be overly simplistic. In order to go beyond the Gaussian regime, we also consider a related non-Gaussian process, which is obtained by considering the BG in polar coordinates. In the isotropic case, the stochastic dynamics of the squared modulus are equivalent to those of the CIR [63] process introduced in finance, which in turn is strictly related to the ABBM model [64], a paradigm for the “crackling noise” in several natural phenomena [65], as well as a “mean field” model for depinning dynamics of disordered elastic manifolds [51]. Since the model can be solved exactly, the analysis of bridge and excursion (which share the same average shape) can be performed analytically [66]. Hereafter, we consider a non-equilibrium extension.

### 6.1. Generic Linear Process

We consider the case of the *d*-dimensional OU process defined as follows:

dx=−α x dt+β dW,

where *x* is a *d* dimensional vector, *W* is a *d* dimensional Wiener process, and α and β are two constant square matrices, the former having eigenvalues with positive real parts and the latter being positive definite. The two conditions are dictated by typical applications and guarantee the existence of a non-degenerate, asymptotic stationary state.

The propagator of the process is a multivariate Gaussian distribution:

(33) $$P\left(x,t|x_{0},t_{0}\right)=\frac{{\left(2\pi \right)}^{-d/2}}{{\left[\det\Sigma (t-t_{0})\right]}^{1/2}}\exp\left\{-\frac{1}{2}\left[x-G\left(t-t_{0}\right)x_{0}\right]\cdot \Sigma^{-1}\left(t-t_{0}\right)\left[x-G\left(t-t_{0}\right)x_{0}\right]\right\},$$

where · is the scalar product between vectors, the matrix G(t) gives the evolution of the mean 〈x(t)〉=G(t)x(0), and Σ(t) gives the covariance of the process

$$\Sigma \left(t\right)=\langle \left[x(t)-\langle x(t)\rangle \right]{\left[x(t)-\langle x(t)\rangle \right]}^{T}\rangle .$$

An explicit computation shows that

(34) $$\begin{array}{ccc} G(t) & = & \exp\left(-\alpha t\right) \end{array}$$

(35) $$\begin{array}{ccc} \Sigma (t) & = & \int_{0}^{t}G\left(t^{\prime }\right)\beta \beta^{T}G^{T}\left(t^{\prime }\right)dt^{\prime }. \end{array}$$

From the last one can be derived the following implicit equation:

$$\Sigma \left(t+\tau \right)=\Sigma \left(t\right)+G\left(t\right)\Sigma \left(\tau \right)G^{T}\left(t\right)$$

Moreover, it is easy to show that the time evolution of G(t) and Σ(t) is ruled by the following equations:

(36) $$\begin{array}{ccc} \frac{d}{dt}G\left(t\right) & = & -\alpha G(t) \end{array}$$

(37) $$\begin{array}{ccc} \frac{d}{dt}\Sigma \left(t\right) & = & -\alpha \Sigma \left(t\right)-\Sigma \left(t\right)\alpha^{T}+\beta \beta^{T}. \end{array}$$

We want to compute the bridge asymmetry defined in Equation (31), which now reads:

$$\Delta \left(x,t\right)=-2\alpha x+\beta \beta^{T}\nabla \log\frac{P(x_{0},t_{f}-t|x,0)}{P(x,t_{f}-t|x_{0},0)}.$$

Now, let us proceed evaluating the $\log\frac{P(x_{0},t_{f}-t|x,0)}{P(x,t_{f}-t|x_{0},0)}$. It turns out that it reads as follows:

$$\frac{1}{2}\left\{\left[x-G\left(t^{\prime }\right)x_{0}\right]\cdot \Sigma^{-1}\left(t^{\prime }\right)\left[x-G\left(t^{\prime }\right)x_{0}\right]-\left[x_{0}-G\left(t^{\prime }\right)x\right]\cdot \Sigma^{-1}\left(t^{\prime }\right)\left[x_{0}-G\left(t^{\prime }\right)x\right]\right\},$$

where $t^{\prime }=t_{f}-t$. Using the usual property of the scalar product ($Mu\cdot v=u\cdot M^{T}v$), the quadratic terms in *x* reduce to

$$\frac{1}{2}x\cdot \left[\Sigma^{-1}\left(t^{\prime }\right)-G^{T}\left(t^{\prime }\right)\Sigma^{-1}\left(t^{\prime }\right)G\left(t^{\prime }\right)\right]x.$$

The gradient of this, using $\nabla \left[x\cdot Mx\right]=\left(M+M^{T}\right)x$, and

$${\left[\Sigma^{-1}\left(t\right)-G^{T}\left(t\right)\Sigma^{-1}\left(t\right)G\left(t\right)\right]}^{T}=\Sigma^{-1}\left(t\right)-G^{T}\left(t\right)\Sigma^{-1}\left(t\right)G\left(t\right),$$

reads

$$\nabla \left\{\frac{1}{2}x\cdot \left[\Sigma^{-1}\left(t^{\prime }\right)-G^{T}\left(t^{\prime }\right)\Sigma^{-1}\left(t^{\prime }\right)G\left(t^{\prime }\right)\right]x\right\}=\left[\Sigma^{-1}\left(t^{\prime }\right)-G^{T}\left(t^{\prime }\right)\Sigma^{-1}\left(t^{\prime }\right)G\left(t^{\prime }\right)\right]x.$$

The linear terms, on the other hand, are

(38) $$\begin{array}{ccc} & & \frac{1}{2}\left[-x\cdot \Sigma^{-1}\left(t^{\prime }\right)G\left(t^{\prime }\right)x_{0}-G\left(t^{\prime }\right)x_{0}\cdot \Sigma^{-1}\left(t^{\prime }\right)x\right. \end{array}$$

(39) $$\begin{array}{ccc} & & \left.+x_{0}\cdot \Sigma^{-1}\left(t^{\prime }\right)G\left(t^{\prime }\right)x+G\left(t^{\prime }\right)x\cdot \Sigma^{-1}\left(t^{\prime }\right)x_{0}\right] \end{array}$$

(40) $$\begin{array}{cc} = & -x\cdot \Sigma^{-1}\left(t^{\prime }\right)G\left(t^{\prime }\right)x_{0}+x_{0}\cdot \Sigma^{-1}\left(t^{\prime }\right)G\left(t^{\prime }\right)x \end{array}$$

(41) $$\begin{array}{ccc} & = & -x\cdot \left[\Sigma^{-1}\left(t^{\prime }\right)G\left(t^{\prime }\right)-G^{T}\left(t^{\prime }\right)\Sigma^{-1}\left(t^{\prime }\right)\right]x_{0} \end{array}$$

whose gradient, since $\nabla \left[x\cdot Mx_{0}\right]=Mx_{0}$, is

$$-\left[\Sigma^{-1}\left(t^{\prime }\right)G\left(t^{\prime }\right)-G^{T}\left(t^{\prime }\right)\Sigma^{-1}\left(t^{\prime }\right)\right]x_{0}.$$

The remaining terms do not depend on *x* and they are wiped out by the $x_{i}$ derivatives in the asymmetry expression Equation (32), which now becomes

(42) $$\begin{array}{c} \Delta \left(x,t_{f}-t\right)=-2\alpha x+\beta \beta^{T}\left\{\left[\Sigma^{-1}\left(t\right)-G^{T}\left(t\right)\Sigma^{-1}\left(t\right)G\left(t\right)\right]x\right.\left.-\left[\Sigma^{-1}\left(t\right)G\left(t\right)-G^{T}\left(t\right)\Sigma^{-1}\left(t\right)\right]x_{0}\right\}, \end{array}$$

Imposing a zero asymmetry for the *d*-dimensional OU process amounts to requiring Δ(x,t)=0 for every *x*, $x_{0}$, and *t*, which gives

(43) $$\begin{array}{ccc} 0 & = & \left[\Sigma^{-1}\left(t\right)G\left(t\right)-G^{T}\left(t\right)\Sigma^{-1}\left(t\right)\right]. \end{array}$$

(44) $$\begin{array}{ccc} 2\alpha & = & \beta \beta^{T}\left[\Sigma^{-1}\left(t\right)-G^{T}\left(t\right)\Sigma^{-1}\left(t\right)G\left(t\right)\right] \end{array}$$

Equation (43) implies the Onsager relations; see for instance Section 5.3.6c of [2], while Equation (44) means that $\Sigma^{-1}\left(t\right)-G^{T}\left(t\right)\Sigma^{-1}\left(t\right)G\left(t\right)$ is independent from *t*, so

(45) $$\Sigma^{-1}\left(t\right)-G^{T}\left(t\right)\Sigma^{-1}\left(t\right)G\left(t\right)=\Sigma^{-1}\left(\tau -t\right)-G^{T}\left(\tau -t\right)\Sigma^{-1}\left(\tau -t\right)G\left(\tau -t\right).$$

On the other hand, the expression for the bridge covariance is

$$\Sigma_{B}^{-1}\left(t\right)=\Sigma^{-1}\left(t\right)+G^{T}\left(\tau -t\right)\Sigma^{-1}\left(\tau -t\right)G\left(\tau -t\right),$$

and Equation (45) implies that $\Sigma_{B}\left(t\right)=\Sigma_{B}\left(\tau -t\right)$, as expected. In Appendix A we compute explicitly the asymmetry Δ for some specific cases of BG in Cartesian coordinates.

### 6.2. Two-Dimensional BG in Polar Coordinates: An Interesting Non-Linear Process

Let now focus on the two-dimensional case, which, without loss of generality, can be parameterized as follows:

(46) $$\alpha =\left(\begin{array}{cc} k_{1} & u-\omega \\ u+\omega & k_{2} \end{array}\right)$$

and

$$\beta =\left(\begin{array}{cc} \sqrt{2T_{1}} & 0\\ 0 & \sqrt{2T_{2}} \end{array}\right).$$

We want to describe the process in polar coordinates, defined as follows:

(47) $$\begin{array}{ccc} v & = & x_{1}^{2}+x_{2}^{2} \end{array}$$

(48) $$\begin{array}{ccc} \tan\theta & = & \frac{x_{2}}{x_{1}}. \end{array}$$

Using Ito’s Lemma, it is easy to derive the sde for the new process (v,θ):

(49) $$\begin{array}{ccc} dv & = & 2\left[T_{1}+T_{2}-v\;\left(k_{1}\cos^{2}\theta +k_{2}\sin^{2}\theta -\;u\sin2\theta \right)\right]dt+ \end{array}$$

(50) $$\begin{array}{ccc} & & \sqrt{8T_{1}v}\;\cos\theta \;dW_{1}+\sqrt{8T_{2}v}\;\sin\theta \;dW_{2} \end{array}$$

(51) $$\begin{array}{ccc} d\theta & = & -\left[\omega +u\cos\left(2\theta \right)+\left(\frac{T_{1}-T_{2}}{v}+\frac{k_{1}-k_{2}}{2}\right)\sin2\theta \right]dt \end{array}$$

(52) $$\begin{array}{ccc} & & -\sqrt{\frac{2T_{1}}{v}}\sin\theta dW_{1}+\sqrt{\frac{2T_{2}}{v}}\cos\theta dW_{2} \end{array}$$

The process v(t),θ(t), at odds with $x_{1},x_{2}$, is not Gaussian, as witnessed by the multiplicative (and singular) diffusion coefficients in the sde.

Note that the equation for dθ is always coupled with *v*. On the other hand, the equation for dv is coupled to θ only when the system is anisotropic, that is, for $T_{1}\neq T_{2}$ (which makes the noise terms correlated) or for anisotropic potential, that is, for $k_{1}\neq k_{2}$ or u≠0.

### 6.3. Mechanical Case

The expression for the asymmetry for a general bridge from $v_{0},\theta_{0}$ to $v_{0},\theta_{0}$ is quite cumbersome. Here, we consider the case of equal temperature $T_{1}=T_{2}=T$, but a non-conservative force, that is ω≠0.

For $T_{1}=T_{2}\equiv T$, the correlation between the noise terms is only apparent, since

(53) $$\begin{array}{ccc} dW_{1}^{\prime } & = & \cos\phi \;dW_{1}+\sin\phi \;dW_{2} \end{array}$$

(54) $$\begin{array}{ccc} dW_{2}^{\prime } & = & -\sin\phi \;dW_{1}+\cos\phi \;dW_{2} \end{array}$$

are two independent Wiener processes, whatever the angle ϕ: $\langle dW_{1}^{\prime }dW_{2}^{\prime }\rangle =0$

In this case, without loss of generality, we can always perform a rotation of the Cartesian coordinates in order to obtain a drift with equal diagonal terms

(55) $$\alpha =\left(\begin{array}{cc} k & u+\omega \\ u-\omega & k \end{array}\right),$$

(here, *u* is the rotated value, not the original value in Equation (46)), since the square modulus *v* is not affected by the rotation, while θ changes by an irrelevant additive constant, which can be absorbed in the initial conditions. In this case, the anisotropy is represented only by *u* and the equation simplifies as follows:

(56) $$\begin{array}{ccc} dv & = & 2\left[2T-v\;(k-\;u\sin2\theta )\right]dt+\sqrt{8Tv}\;dW_{1} \end{array}$$

(57) $$\begin{array}{ccc} d\theta & = & -\left[\omega +u\cos(2\theta )\right]dt+\sqrt{\frac{2T}{v}}dW_{2}. \end{array}$$

We call this case the “mechanical Brownian gyrator” because the origin of the probability current is only in the forces (ω≠0) and not in the anisotropy of the thermal baths.

It is easy to show that the distribution and the propagator of the process v,θ can be expressed in terms of the Gaussian propagator of the process in Cartesian coordinates with average $x_{0}G\left(t\right)$ and covariance Σ(t), as given previously, where now the initial condition coordinates of $x_{0}$ are $\sqrt{v_{0}}\cos\left(\theta_{0}\right)$ and $\sqrt{v_{0}}\sin\left(\theta_{0}\right)$:

(58) $$\begin{array}{ccc} P_{pol}\left(v,\theta ,t\right) & = & \frac{1}{2}P_{cart}\left(x_{1}=\sqrt{v}\cos\theta ,x_{2}=\sqrt{v}\sin\theta \right) \end{array}$$

(59) $$\begin{array}{ccc} P_{pol}\left(v,\theta ,t|v_{0},\theta_{0}\right) & = & \frac{1}{2}P_{cart}\left(x_{1}=\sqrt{v}\cos\theta ,x_{2}=\sqrt{v}\sin\theta ,t|x_{0}\left(v_{0},\theta_{0}\right)\right) \end{array}$$

(with the factor 1/2 coming from the Jacobian $dx_{1}dx_{2}=\frac{1}{2}dvd\theta )$.

Using Equation (32), the bridge asymmetry for the process v(t),θ(t) in the isotropic case (u=0) takes a very simple form:

(60) $$\Delta_{iso}=\left(\begin{array}{c} \frac{2k\sqrt{vv_{0}}}{\sinh(kt)}\sin\left(\theta_{0}-\theta \right)\sin\left(\omega \;t\right)\\ -2\omega -\sqrt{\frac{v_{0}}{v}}\frac{k}{\sinh(kt)}\cos\left(\theta_{0}-\theta \right)\sin\left(\omega \;t\right) \end{array}\right).$$

Considering the case of the bridge from the origin to the origin, that is, for $v_{0}=0$ (the value of $\theta_{0}$ is irrelevant at the origin), the asymmetry reduces to

(61) $$\Delta_{iso}^{O}=\left(\begin{array}{c} 0\\ -2\omega \end{array}\right).$$

Note that in this case, the asymmetry for the *v* component is zero, despite the dynamics being out of equilibrium. This can be understood by considering the sde of the process in the isotropic case u=0:

(62) $$\begin{array}{ccc} dv & = & 2\left(2T-kv\right)dt+2\sqrt{2Tv}\;dW_{1} \end{array}$$

(63) $$\begin{array}{ccc} d\theta & = & -\omega \;dt+\sqrt{\frac{2T}{v}}\;dW_{2}. \end{array}$$

As a consequence of the isotropy of the drift term, the equation for the squared modulus *v* does not depend on θ (while θ is a non-Markovian process slaved to the process *v*).

As previously mentioned, Equation (62) describes a CIR process [63] r(t) under an appropriate choice of parameters:

$$dr=\alpha \left(\beta -r\right)\;dt+\sigma \sqrt{r}\;dW,$$

which is rigorously equivalent to an ABBM process [66].

Due to this partial decoupling, the *v* component of the stationary current is zero. This can be shown considering the expression of the propagator of the process

(64) $$P_{pol}\left(v,\theta ,t|v_{0},\theta_{0}\right)=\frac{k}{4\pi T(1-e^{-2kt})}\exp\left(-\frac{k\left[v+v_{0}e^{-2kt}-2e^{-kt}\sqrt{vv_{0}}\cos\left(\theta_{0}-\theta +\omega t\right)\right]}{2T(1-e^{-2kt})}\right)$$

from which the stationary distribution of can be computed as the asymptotic limit:

$$P_{pol}^{st}\left(v,\theta \right)\equiv \lim_{t\to \infty }P_{pol}\left(x,\theta ,t|v_{0},\theta_{0}\right)=\frac{k}{4\pi T}\exp\left[-\frac{kv}{2T}\right].$$

Note that $P_{pol}^{st}$ does not depend on θ. Using the expression for $P_{pol}^{st}$, one obtains the stationary velocity current:

(65) $$V_{s}=\left(\begin{array}{c} 0\\ -\omega \end{array}\right)$$

So, while the full process has a non-zero current, the restricted process v(t) is a Markovian process with zero current (hence satisfying detailed balance). The propagator of the v(t) process can be computed marginalizing the full propagator $P_{v}\left(v,t|v_{0},\theta_{0}\right)\equiv \int d\theta P_{pol}\left(v,\theta ,t|v_{0},\theta_{0}\right)$ that does not depend on $\theta_{0}$, as follows:

$$P_{v}\left(v,t|v_{0}\right)=\frac{k}{4T}\left(1+\coth(kt)\right)\exp\left\{-\frac{k}{4T}\left[v-v_{0}+\left(v+v_{0}\right)\coth\left(kt\right)\right]\right\}I_{0}\left(\frac{k\sqrt{vv_{0}}}{2T\sinh(kt)}\right),$$

where $I_{0}$ is the modified Bessel function of the first kind of index 0.

Coming back to the general anisotropic case, we consider the bridge from the origin to the origin, that is, $v_{0}=0$, and we obtain for the asymmetry the following expression:

(66) $$\Delta^{O}=\left(\begin{array}{c} u\;\omega \;v\cos\left(2\theta +\psi \right)D_{1}\left(t\right)\\ -2\omega \left[1-u\sin\left(2\theta +\psi \right)D_{2}\left(t\right)\right] \end{array}\right)$$

where

$$\cos\psi =\frac{k}{\sqrt{k^{2}+\omega^{2}}}$$

and

$$\begin{array}{ccc} D_{1} & = & \frac{4}{\sqrt{k^{2}+\omega^{2}}}\left(\frac{2k^{2}\left(k^{2}-u^{2}+\omega^{2}\right)\sinh^{2}\left(\left(t_{f}-t\right)\sqrt{u^{2}-\omega^{2}}\right)}{D_{3}}+1\right)\\ D_{2} & = & \frac{\sqrt{k^{2}+\omega^{2}}}{D_{3}}\left\{k^{2}\left(-\cosh\left(2\left(t_{f}-t\right)\sqrt{u^{2}-\omega^{2}}\right)\right)+\right.\\ & & +\left.\left(u^{2}-\omega^{2}\right)\cosh\left(2k\left(t_{f}-t\right)\right)+k^{2}-u^{2}+\omega^{2}\right\}\\ D_{3} & = & k^{2}u^{2}\cosh\left(2\left(t_{f}-t\right)\sqrt{u^{2}-\omega^{2}}\right)+\\ & & +\left(k^{2}+\omega^{2}\right)\left(\omega^{2}-u^{2}\right)\cosh\left(2k\left(t_{f}-t\right)\right)-\omega^{2}\left(k^{2}-u^{2}+\omega^{2}\right) \end{array}$$

It easy to see that $\Delta^{O}$ in Equation (66) recovers the isotropic case for u=0.

### 6.4. Thermal Case

We briefly discuss the much more complicated case in which ω=0, but $T_{1}\neq T_{2}$. The computations in this case rapidly become cumbersome, so we restrict our discussion to the case

$$A=\left(\begin{array}{cc} k & u\\ u & k \end{array}\right)$$

where the potential is anisotropic but the axis of the elliptic equipotential lines are oriented to ±π/4 in the Cartesian axes.

The expression for the asymmetry Δ is too long to be shown here, but we can write out the expression for $v_{0}=0$ and small values of $dT=T_{2}-T_{1}$

$$\Delta^{O}=\left(\begin{array}{c} \frac{2v\cos\left(2\theta \right)\left[k^{2}\left(\cosh\left(2u\left(t-t_{f}\right)\right)-1\right)+u^{2}\left(1-\cosh\left(2k\left(t-t_{f}\right)\right)\right)\right]}{\left[\cosh\left(2k\left(t-t_{f}\right)\right)-\cosh\left(2\left(t-t_{f}\right)u\right)\right]kT_{1}}dT+O\left(dT^{2}\right)\\ \frac{\left(k^{2}-u^{2}\right)\sin\left(2\theta \right)+k\cosh\left(2u\left(t-t_{f}\right)\right)\left(u+k\sin\left(2\theta \right)\right)-u\cosh\left(2k\left(t-t_{f}\right)\right)\left(k+u\sin\left(2\theta \right)\right)}{\left[\cosh\left(2k\left(t-t_{f}\right)\right)-\cosh\left(2\left(t-t_{f}\right)u\right)\right]kT_{1}}dT+O\left(dT^{2}\right) \end{array}\right)$$

while for small *u*,

$$\Delta^{O}=\left(\begin{array}{c} \frac{v\sin\left(2\theta \right){\left(T_{1}-T_{2}\right)}^{2}u}{T_{1}T_{2}}+O\left(u^{2}\right)\\ \frac{\left(T_{1}-T_{2}\right)\left(-T_{1}+\cos\left(2\theta \right)\left(T_{1}-T_{2}\right)-T_{2}\right)u}{2T_{1}T_{2}}+O\left(u^{2}\right) \end{array}\right).$$

Note that for u=0 (isotropic potential), the asymmetry is zero, since the two degrees of freedom are uncoupled, and each one equilibrates with the respective thermal bath.

## 7. Conclusions

We have summarized the core elements for constructing a theory of stochastic bridges and their time-reversal statistics, aiming to derive a general formula that quantifies the asymmetry under time reversal in avalanche-like fluctuations. This asymmetry holds potential for inferring thermodynamic properties from experimental data. Indeed, entropy production, arising from external forcing and energy dissipation in physical systems, manifests in fluctuations as a violation of detailed balance, as consistently observed as an asymmetry in the stochastic bridges we analyzed. Our primary result, Equations (31) and (32), suggests the possibility of a cancellation between terms, even when time-reversal symmetry is broken, although we have yet to encounter such examples. Such a formula, when applied to a Brownian gyrator in a generic dimension, Equation (42), demonstrates that the symmetry of a bridge connecting the origin with itself always corresponds to the condition of detailed balance. As a non-Gaussian process, the BG in d=2 was analyzed in polar coordinates. The problem was split into two cases: the mechanical case (equal temperatures and an applied external torque) and the thermal case (no torque but different temperatures). In the mechanical case, the asymmetry of the polar bridge connecting the origin with itself takes the form given in Equation (66), which is partially symmetric (the magnitude component is zero) in the case u=0, even when detailed balance is broken. In the thermal case, on the contrary, the expression for the asymmetry has been made explicit for small departures from equilibrium. At first order, these expressions can become empty only when detailed balance is satisfied (equal temperatures), but no rigorous conclusions are available at this moment.

Finally, we emphasize that “partial information” implies not only limited observability of the asymmetry’s components but also a restriction of the bridge’s definition to a subspace of initial and final conditions. This constraint arises because the boundary conditions can only be imposed on the observable degrees of freedom. The exploration of “partial bridges”—trajectories initiating and terminating within a subspace rather than at single points—will be a subject of our future research.

## Data Availability

Data are contained within the article.

## Appendix A. Brownian Gyrator in Cartesian Coordinates

Here, we derive explicitly the asymmetry of the bridge for the Brownian gyrators in Cartesian coordinates. We limit ourselves to the cases in which the computation can be expressed in simple analytical expressions.

### Appendix A.1. Brownian Gyrator

Without loss of generality, we consider the matrix β as a diagonal matrix with diagonal elements equal to $\sqrt{2T_{1}}$ and $\sqrt{2T_{2}}$, that is

(A1) $$B=\left(\begin{array}{cc} 2T_{1} & 0\\ 0 & 2T_{2} \end{array}\right)$$

The drift matrix can be parameterized as the sum of a symmetric and an anti-symmetric matrix $A=A_{s}+A_{a}$:

(A2) $$A_{s}=\left(\begin{array}{cc} k_{1} & u\\ u & k_{2} \end{array}\right)$$

and

(A3) $$A_{a}=\left(\begin{array}{cc} 0 & \omega \\ -\omega & 0 \end{array}\right).$$

The equilibrium case is obtained for $T_{1}=T_{2}$ and ω=0. When one of these conditions does not hold, the process is not in equilibrium.

### Appendix A.2. Mechanical Brownian Gyrator

We consider the case in which the matrix β is proportional to the identity β=TI, that is $T_{1}=T_{2}=T$, and the forces are isotropic, that is

$$\alpha =\left(\begin{array}{cc} k & \omega \\ -\omega & k \end{array}\right)$$

and

$$\beta =\left(\begin{array}{cc} \sqrt{2T} & 0\\ 0 & \sqrt{2T} \end{array}\right)$$

In this case, one can easily compute the expressions for Δ that read as follows:

(A4) $$\Delta =2\left(\begin{array}{c} x_{2}\;\omega -x_{2}^{0}\;k\frac{\sin\left(\omega \left(t-t_{f}\right)\right)}{\sinh\left(k\left(t-t_{f}\right)\right)}\\ -x_{1}\;\omega +x_{1}^{0}\;k\frac{\sin\left(\omega \left(t-t_{f}\right)\right)}{\sinh\left(k\left(t-t_{f}\right)\right)} \end{array}\right)$$

that vanish for ω=0, while for $x_{0}=0$

(A5) $$\Delta =2\omega \left(\begin{array}{c} x_{2}\\ -x_{1} \end{array}\right)$$

The anisotropic case, where

$$\alpha =\left(\begin{array}{cc} k & u+\omega \\ u-\omega & k \end{array}\right)$$

can also be easily computed. Here, we write the result to first order in ω:

(A6) $$\Delta =2\left(\begin{array}{c} \frac{\left(k^{2}-u^{2}\right)x_{1}-k\cosh\left(2u\left(t-t_{f}\right)\right)\left(kx_{1}+ux_{2}\right)+u\cosh\left(2k\left(t-t_{f}\right)\right)\left(ux_{1}+kx_{2}\right)}{ku\left(\cosh\left(2k\left(t-t_{f}\right)\right)-\cosh\left(2u\left(t-t_{f}\right)\right)\right)}\omega +O\left(\omega^{2}\right)\\ -\frac{\left(k^{2}-u^{2}\right)x_{2}-k\cosh\left(2u\left(t-t_{f}\right)\right)\left(ux_{1}+kx_{2}\right)+u\cosh\left(2k\left(t-t_{f}\right)\right)\left(kx_{1}+ux_{2}\right)}{ku\left(\cosh\left(2k\left(t-t_{f}\right)\right)-\cosh\left(2u\left(t-t_{f}\right)\right)\right)}\omega +O\left(\omega^{2}\right) \end{array}\right).$$

The general case for the drift matrix

$$\alpha =\left(\begin{array}{cc} k_{1} & u+\omega \\ u-\omega & k_{2} \end{array}\right)$$

can be solved using the above solutions provided a coordinate rotation is applied to *x*, which corresponds to a rotation of the asymmetry vector Δ.

### Appendix A.3. Thermal Brownian Gyrator

Here, we consider the case ω=0, $T_{1}=T$, but $T_{2}=T+dT$. The general expression is quite cumbersome. For $k_{1}=k_{2}=k$ and $x_{0}=0$ and small dT, it reads as follows:

(A7) $$\Delta =\left(\begin{array}{c} -\frac{u}{kT}\left(\frac{2\left(u^{2}-k^{2}\right)x\sinh^{2}\left(u\left(t_{f}-t\right)\right)}{u\left(\cosh\left(2k\left(t-t_{f}\right)\right)-\cosh\left(2u\left(t_{f}-t\right)\right)\right)}+ux_{1}+kx_{2}\right)dT+O\left({\mathrm{dT}}^{2}\right)\\ \frac{u}{kT}\left(\frac{2\left(u^{2}-k^{2}\right)y\sinh^{2}\left(u\left(t_{f}-t\right)\right)}{u\left(\cosh\left(2k\left(t-t_{f}\right)\right)-\cosh\left(2u\left(t_{f}-t\right)\right)\right)}+kx_{1}+ux_{2}\right)dT+O\left({\mathrm{dT}}^{2}\right) \end{array}\right).$$
